# Autointoxication

**Published:** 1905-10

**Authors:** D. L. Field


ATLANTA

Journal-Record of Medicine.

Successor to Atlanta Medical and Surgical Journal, Established 1855, and Southern Medical Record, Established 1870.

OWNED BY THE ATLANTA MEDICAL JOURNAL CO.

Published Monthly.

VOL. VII. OCTOBER, 1905. No. 7.

BERNARD WOLFF, M.D., EDITOR, Nos. 319–20 Prudential.

M. B. HUTCHINS, M.D., BUSINESS MANAGER, 1007–1008 Century Bldg.

E. G. BALLENGER, M.D., ASSOCIATE EDITOR AND ASSISTANT MANAGER.
J. N. LECONTE, M.D., FOREIGN CORRESPONDENT.

### *ORIGINAL COMMUNICATIONS.*

### AUTOINTOXICATION.*

BY D. L. FIELD, M.D.

To attempt to present anything to the learned and experienced physicians before me that will enlighten them or refresh their memories of things they long ago learned is a task I shrink from undertaking; but in racking my brain to hit upon something that has not been worn threadbare by frequent iteration and reiteration, I shall try to present a few facts and theories upon the theme above stated.

The condition known as autointoxication is not so thoroughly studied perhaps, nor so well understood, but that it may very profitably be considered both from a physiological and pathological point of view; and I freely confess that I have not been such a close student and observer, nor had that experience, chemical and microscopic research, that would warrant me in attempting to evolve anything unaided by reference to eminent bacteriologists

*Read before Mississippi Valley Medical Association, Indianapolis, Ind.

and physiologists. What I *shall* attempt to present will be largely gleanings from the writings of eminent men in the profession.

What is autointoxication?

Bouchard says it is "self-poisoning of the individual." To my mind that is not sufficiently definite, as it implies that it is a condition man may bring upon himself wilfully or ignorantly, when in reality autointoxication is due to what would seem to be an unavoidable physiological process; in other words, putrefactive processes in the alimentary canal, and the development of physiological and pathological alkaloids, which play an important part in the production of disease—independent of any knowledge or control of the individual.

An author says: "Considering the multiform changes that take place in the small intestines during the process of digestion—changes of a chemical, putrefactive and fermentative nature—there must be produced substances of a highly complex nature, alkaloids or ptomaines, which, when absorbed into the system, may seriously affect the vitality of the individual."

We are all more or less protected against the injurious effects of micro-organisms by the fact that products secreted as the result of microbic activity react upon the organisms themselves, and thus limit their longevity. It is the function of the gastro-intestinal juices, aided by the movements of the stomach and intestines, to convert foods into such soluble forms that they can be utilized by the economy. Babes has isolated from the normal intestinal mucus five species of bacteria, while an enormous number of micro-organisms exist in the large intestines and feces. All the organisms resist the action of the digestive fluids, save a few which are dissolved in the gastric juice.

Bouchard says: "A man is constantly on the edge of a precipice; he is continually on the threshold of disease; every moment of his ife he runs the risk of being overpowered by poisons generated within him."

Self-poisoning is only prevented by the activity of the excretory organs, chiefly the kidneys, and by the watchfulness of the liver, which acts the part of a sentinel to the materials brought to it by the portal vein from the alimentary canal. Disease is something not altogether apart from the individual, but the two are often found living in identical conditions.

Chemical investigation has shown how disease depends upon the products of putrefaction and fermentation, rather than by the direct action of microbes upon the system. It is *this* fact which renders the life-history of bacteria so valuable to us; for long after the microbes have been destroyed the enzymes or ferments which they formed continue to act, and are not destroyed by a temperature which is destructive to the organisms themselves. This search-light thrown upon microbic, putrefactive and fermentative conditions in the alimentary canal *must* be of incalculable value as confirmatory of the antiseptic treatment of enteric fever.

An author says: "It is only lately that we have come to recognize that once the dangers incident to typhoid fever have been successfully overcome, there are risks still to be met; in a word, autointoxication from poisons generated in the intestinal canal."

There are few medical men, however, who have not had some experience of success who have followed the antiseptic treatment ot typhoid fever.

Oliver says: "The part played in *mental* diseases by autointoxication is attracting wide attention. Maniacal urine gives rise to excitement and convulsions when injected into an animal, while the injection of urine from a case of melancholia is followed by depression ot spirits, restlessness and stupor—a proof that autointoxication is the cause and not the effect of the mental state."

Besides the mental disorders that arise during the course of such infectious diseases as typhoid and emptive fevers, there is little doubt that these disturbances are due to the action of pathogenic organisms, either directly or indirectly, through the influence of their toxines; and according to the stages of the illness, so is the character of the mental symptoms. One has but to mention these facts to throw into bold relief the excellent results that follow the faithful administration of antiseptics and excitants of the emunctories.

Again Bouchard says: "Substances the most essential to the constitution of the body may become hurtful when they accumulate. If the subtraction of water is dangerous, its excess is no less so; it changes the conditions of osmosis; it causes a swelling up of the cells, and washes out their dialysable material; it thus disturbs their chemical composition, and weakens or prevents their functional activity. Mineral substances can by their excess equally determine accidents that are truly toxic—the salts of potassium especially. The most important excrementitious material—carbonic acid—could not be retained in the organism a few minutes without death being the result. The biliary acids, also, if they do not find an escape outwardly, produce fatal results. All the soluble ferments elaborated by certain glands can exercise a poisonous influence, either local or general. We have in certain secretions, the saliva for example, products extremely toxic, and which are not ferments. This toxicity is only partly due to alkaloids. Whatever opinion we may have in regard to the origin of alkaloids, it is certain that we meet with them in normal tissue, and it is possible that they may be one of the results of this disassimilation of animal cells; without multiplying examples, the augmentation of normal substances, either by increased formation or retention, can induce quite a series of toxic accidents, some of which may be named as asphyxia, uremia, cholemia and glycemia. Perverted nutrition leads up to the development of new substances which may become toxic. There are often formed in the organism peptones which do not originate in the intestinal tube, but which are injurious in this sense, that being dialysable, they escape by the urine, and thus bring about an abnormal spoliation of the organism. There are thus produced abnormal albumens, which escape by the kidneys, and seem capable of destroying the nutrition of the renal epithelium, and inducing certain nephrites. The organism, it would seem, therefore is both in the normal and pathological state, the receptacle and laboratory of poisons.

To repeat: The poisons which rank first, are mineral substances introduced with our food; then come the products of physiological secretion—saliva and bile—the products of digestion; digestion too, while it transforms albuminoid substances into peptones also gives birth to alkaloidal poisons; and lastly, toxic substances resulting from intestinal putrefaction. Without doubt, the stools eliminate the greater part of these poisons, which are expelled with them, but nevertheless, owing to the slow movement of the intestinal contents, the mucous membrane absorbs a certain part of them. We find in the close relationship of our tissues, other poisons which are the result of the life of cells. They pass out into the extra cellular fluids, along which they pass into the lymphatics and blood-vessels. It is therefore, into the *blood* that all the poisons are carried, the whole of those made by the tissues, and a part of those formed in the digestive tube.

Bouchard again says: "The blood is continually being traversed by a current of toxic material. It is true the poison is never found in it except in harmless quantities. There is less toxic matter in the blood than in the organs. The anatomical elements form substances which, if retained, would fetter their life, but substances leave them little by little, in order to penetrate into the blood. The quantity of toxic material eliminated by the kidneys in twenty-four hours is, without doubt, one-half of what is necessary to kill the whole of the body, and the blood has really received that quantity in twenty-four hours; but the elimination is incessant, and at every instant of the day the blood never contains at one time, more that an infinitely small fraction of poison.

Is bile toxic? Bouchard asserts that the biliary salts are toxic in infinitesimal quantities. They do not kill by direct intoxication; we can see by the microscope the harm they do. They dissolve and break up the blood globules, and also other cells; striated muscular fibers and the cells of the liver. They therefore cause anatomical lesions, and intoxication from setting free substances that are toxic which enter into the composition of the cellular elements. This intoxication develops but slowly; so long as there is functional activity of the kidneys, all goes well; but if not, they die by the intoxication of the biliary salts and by other products of cellular destruction. If bile is toxic, directly or indirectly, the intestines already are, so far as the bile is concerned, a source of intoxication in as feeble a proportion as we please, but really so. From these pass on into the blood, in addition, other materials, which are eliminated by the urine, such as mineral substances derived from the blood, and other toxic matter of alimentary origin.

Yet this is only a minimum portion of the poisons which the blood may derive from the intestinal canal. The blood is intoxicated from intestinal putrefaction, not only that which arises from imperfect metamorphosis of digested matter, but that which the presence of micro-organisms in the intestinal tube incessantly maintain. In the digestive canal, the most favorable conditions for the elaboration of poisons are realized. There are found nitrogenous substances, already peptonized; and peptones are, as is well known, excellent culture media for microbes. They are in constant association with water in a tube at a temperature of thirty-seven degrees; the intestinal canal is constantly open exteriorly. Besides, the foods taken carry in with them putrefactive agents, respiration allows of the deposition of dust in the pharanx, which, with the act of deglutition, is caught by the saliva along with the micro-organisms which it conceals. The conditions favorable for the maintenance of putrefaction are so numerous, that we may wonder whether digestion ever can go on normally? It has been supposed that the gastric juice would arrest all fermentation; but infectious agents have not been destroyed in the stomach—only neutralized, or passed into a state of latent vitality. The action of organized ferments recommences when the food has passed the pylorus. The *bile* has been regarded as arresting fermentation; but the bile is capable of undergoing fermentation—if not putrefaction. It can therefore but feebly oppose fermentation in the intestines, and, particularly, the large intestines, capable of passing products of putrefaction into the *blood!* To sum up, the toxic substances found in the intestinal canal are the salts of potassium and ammonia, the bile, and the residues of putrefaction.

Bouchard states that the toxic products coming from the tissues, secreting organs, foods, and putrefaction, introduced into the blood, impose these poisons *principally* on the *renal* emunctories. The blood, therefore, is not habitually toxic, because the urine is strongly so. If it were not so, the blood would be heavily charged with poisons. In a normal condition of the kidneys, the accidents of intoxication are not observed. To all cases of death arising from suppression of the renal function, we apply the term, *uremia;*

but what we already *know* enables us to foresee a complexity of phenomena hidden under this name.

Now if the body is the receptacle and laboratory of poisons, which are so constantly endangering health and life, the question would naturally be suggested—How is the system protected from early destruction? One author says he is *astonished* to find so many poisons in the intestinal canal, and yet so few toxic accidents? And considering that poisons of the intestinal canal are innocuous to the animal which has formed them, while the same poisons become harmful to an animal of any other species, into which they have been introduced by the rectum or stomach, he is led to think that each kind of animal has the power of destroying of itself the poisonous substances which it forms.

Hoffmiester asserts that "the white globules play a part in the transportation of peptones into albumen, since we no longer find in the emunctories the peptones which we have injected into the blood." The protective action of the liver is invoked to neutralize the poisons; this organ stops, arrests, as we know, certain portions of our food; it impedes the passage of grape sugar, and stores up glucose under the form of glycogen. It plays a protective part in arresting alkaloidal substances. Perhaps the liver itself acts in opposition to all the poisons of the organism, and robs it of the blood which carries them. Schiff introduced a quantity of nicotine into the intestinal canal of an uninjured animal, and it was not found to be intoxicated by it. The same dose intoxicated the animal if the portal vein is ligatured, for then the toxic substance reaches the general circulation by the accessories of the portal venous system, without having passed through the liver, which would have arrested it. The part played by the intestinal emunctories in the elimination of certain poisonous substances is attested by the commonly feted stools of persons who frequent the post mortems. The feted character recalls the putrid odor of the emanations from the cadaver. Schiff claims that the liver arrests poisonous substances, and restores them to the intestines, or transforms them into harmless matter.

Bouchard says: "The urine does *not* carry off *all* the poisonous matter secreted by the liver; therefore, the greater part of this substance *must* be neutralized in some *other* part of the body." This neutralization is affected in the intestines, in the liver, in the tissues, and in the blood."

Schiff thinks the bile does not poison us, because the liver takes it back and rejects it, to take it back again, and reject it; and that each time a smaller and smaller portion is absorbed.

Bouchard thinks the *tissues* play a protective part in consuming and transforming the bile, which, having been absorbed, has penetrated the general circulation.

Therapeutically then, what shall be done? The first thing would be to prevent, or *limit*, their penetration into the general circulation. If they have been absorbed, we must try to destroy them.

We have found that the liver has the power of arresting poisons; it withdraws them from the intestines, and eliminates, or destroys them! We should, therefore, stimulate its action by proper therapeutic agents. If the poisons have escaped the liver, they should be eliminated by the skin, the lungs; the intestines, and the kidneys. If all these attempts fail, we should have recourse to certain antidotes, which tend to counteract the physiological effects of the poisons which menace the system. We have a striking example of the antagonistic properties of poisons, in atropine and pilocarpine!

An author says: "We must never neglect, in autointoxication, to keep up the strength of the patient, so that he may have time to eliminate the poisons; sometimes we only need to keep him alive a *few minutes* more in order to save him; we can not provide him with radical force, but he requires *active* force; thus we are led to administer *stimulants*, which may awaken the forces remaining latent. Therapeutic treatment is effected to a certain extent by nature; or a portion of it is carried out by our organs; in uremia, one of the poisons is already lessened—disassimilation being checked by the disease itself in uremic and other patients attacked with self-poisoning.

Poisoning by substances of *alimentary* origin may be diminished.

If potass. really kills, the diminution of solid ingesta diminishes the poisoning; now those patients *drink*—but no longer eat. They must not take broths that contain the mineral elements of meat. Finally, the course to be pursued is to restrict diet; stimulate all the functions of the body by an active and intelligent therapeutic treatment.

The conclusions of Bouchard is that the effects of self-poisoning can be largely controlled by an *antiseptic* treatment, as he contends that *such* a course destroys the alkaloids in the fecal matter and the urine and diminishes the toxicity of all poisons generated in the body.

Before concluding this paper, and as illustrating the violent nature of toxins formed from fecal putrefaction and bilious fermentation, I shall mention the case of my grandson, aged seven years and four months; who was taken suddenly ill with high fever, highly flushed face, hot, dry skin, and nervous jactitations. A few hours after these symptoms manifested themselves, I reached his bedside, and immediately prescribed antipyretics, antispasmodics, and ordered a copious enema of warm soap and water. I should have stated that he complained of a severe "all-round" headache. I gave phenacetine and hydrotherapy, etc.

I had not gone from the house a half-hour till I was hurriedly summoned back, as the patient was seized with a violent epileptiform convulsion. So soon as he recovered consciousness I administered a brisk cathartic, and ordered five grain doses of bromide of sodium, and one drop doses of fluid extract gelsemium, to be repeated every hour, till three doses were given; then to be given every three hours. Cold applications to the head and hot water bags to the feet were used. If jactitation recurred, a handkerchief with chloroform was held to his nose till twitching ceased. Under the influence of constant bathing, and the antispasmodics above named, he soon became tranquil, and slept; but the high pyrexia and hot head continued. In the evening, as a result of a large dose of oil, his bowels began to act, and the discharges were greenish-yellow, and exceedingly offensive. After four such motions, his fever rapidly declined, and after midnight that night, he was free of fever, and to all appearances, well. The following morning he was up and about, as though he had never been ill.

The case clearly exemplifies the correctness of the theory of what frightful mischief indigestible materials, when in a state of putrefaction, may exert on the nervous system, rapidly endangering life—the fever, nausea, and nervousness being entirely symptomatic; the cause being the accumulation of poisons in the alimentary canal from putrefaction of *ingesta,* and bile. Evidently, the thing to do was to promptly avert spasm, and clean out the canal. To avert spasm, I don't think anything excels bromides and gelsemium; and for quick cathartics, give a double portion of castor oil—preceded by copious flushing of the bowels. There was no time for the slow action of the intestinal antiseptics, and further more, they could do no good in the intestines loaded with contents which had undergone poisonous fermentation and putrefaction.

Another case illustrating intestinal poisoning ; a case of typhoid fever, fourth week, with high pyrexia and delirium, in which I gave an enema every morning, composed of a drachm of turpentine mixed in the yolk of an egg beaten up, and added to a quart of warm water, which was stirred well before administering. It prevented tympanites, and the consequent danger from perforation, which, by the way, I believe to be the chief cause for such a result.

One morning the nurse phoned me that my patient had a temperature 107 degrees, and a pulse almost beyond being counted. I hurried to his bedside, and ordered a half gallon of warm normal salt solution, which I slowly injected into the bowels, and it was soon followed by a copious action, which contained over a pint of black coagulum and sloughs from necrosed tissue. In six hours thereafter, his temperature had fallen to 102 degrees and pulse to 99. His head cleared up considerably, his nervousness greatly abated, and he slept.

Never after did he have a *high* temperature, or *low* delirium.

Certainly this was a case where death was imminent from the pressure of putrescent matter in the small intestines. Had such material been allowed to remain, fatal poisoning would have been inevitable.

